# Virological efficacy of PI monotherapy for HIV-1 in clinical practice

**DOI:** 10.1093/jac/dkw265

**Published:** 2016-07-07

**Authors:** Kate El Bouzidi, Dami Collier, Eleni Nastouli, Andrew J. Copas, Robert F. Miller, Ravindra K. Gupta

**Affiliations:** 1 Research Department of Infection, Division of Infection and Immunity, University College London, London, UK; 2 Research Department of Infection and Population Health, University College London, London, UK; 3 Department of Clinical Virology, University College London Hospitals NHS Trust, London, UK; 4 NIHR University College London Hospitals Biomedical Research Centre, London, UK; 5 Department of Clinical Research, Faculty of Infectious and Tropical Diseases, London School of Hygiene and Tropical Medicine, London, UK

## Abstract

**Background:**

Clinical trials of PI monotherapy indicate that most participants maintain viral suppression and emergent protease resistance is rare. However, outcomes among patients receiving PI monotherapy for clinical reasons, such as toxicity or adherence issues, are less well studied.

**Methods:**

An observational study of patients attending an HIV treatment centre in London, UK, who had received PI monotherapy between 2004 and 2013, was conducted using prospectively collected clinical data and genotypic resistance reports. Survival analysis techniques were used to examine the times to virological failure and treatment discontinuation.

**Results:**

Ninety-five patients had PI monotherapy treatment for a median duration of 126 weeks. Virological failure occurred during 64% of episodes and 8% of patients developed emergent protease mutations. We estimate failure occurs in half of episodes within 2 years following initiation. Where PI monotherapy was continued following virological failure, 68% of patients achieved viral re-suppression. Despite a high incidence of virological failure, many patients continued PI monotherapy and 79% of episodes were ongoing at the end of the study. The type of PI used, the presence of baseline protease mutations and the plasma HIV RNA at initiation did not have a significant impact on treatment outcomes.

**Conclusions:**

There was a higher incidence of virological failure and emerging resistance in our UK clinical setting than described in PI monotherapy clinical trials and other European observational studies. Despite this, many patients continued PI monotherapy and regained viral suppression, indicating this strategy remains a viable option in certain individuals following careful clinical evaluation.

## Introduction

PI monotherapy is an appealing option in a subset of patients for whom combination ART (cART) is unsuitable for clinical reasons, such as adverse effects associated with nucleoside reverse transcriptase inhibitors, interactions with other medication and adherence issues. Combination regimens may be more difficult to adhere to because of reduced tolerability, increased pill burden or demands such as the timing of doses. Theoretically, modern boosted PIs are good candidates for use as monotherapy as this class is known to have a high genetic barrier to resistance following viral failure, requiring several mutations before phenotypic drug susceptibility is significantly reduced.^1,2^ In an era when patients will initiate treatment earlier and can expect to be taking therapy for decades, other potential advantages of PI monotherapy include the avoidance of toxicity and preservation of long-term treatment options.^3,4^

Randomized controlled trials investigating PI monotherapy as a maintenance strategy indicate that most participants maintain viral suppression and the emergence of resistance-associated protease mutations is rare. Trials showed a wide range of virological failure when a threshold of plasma HIV RNA >50 copies/mL was used, varying from 5% to 53% over 48 or 96 week follow-up periods.^5–12^ In PIVOT, the largest PI monotherapy trial to date, which included 296 patients in the monotherapy arm, 31% experienced virological failure.^5^ In the MONARK trial,^13^ the only PI monotherapy trial of ART-naive patients who were viraemic at baseline, the proportion that did not achieve sustained virological suppression was 33% by week 48 and 54% by week 96. Most trial protocols mandate a switch to cART in the case of virological failure and so there is little information on virological re-suppression on monotherapy. In the PIVOT study, of the 93 patients with virological rebound, 22 had spontaneously re-suppressed on subsequent testing and a further two re-suppressed after changing the PI but remaining on monotherapy.^5^ There were considerable differences among trials in the definition of emergent mutations, with some reporting all protease mutations and others only the mutations related to the specific PI agent used for monotherapy. The proportion of trial participants that developed emergent protease mutations when PI monotherapy was used as a maintenance strategy was on average <1% (range 0%–2.3%).^5–12,14,15^ Emergent resistance resulted in the loss of future PI options in 1% of patients in the PIVOT trial.^5^ There was a higher proportion of emergent protease mutations, 6%, in the MONARK trial, where PI monotherapy was used as first-line therapy.^13^

However, the results of these trials may not be generalizable to patients who receive PI monotherapy for clinical reasons. Trial participants had mostly already achieved virological suppression, did not have pre-existing protease resistance mutations and presumably were considered likely to adhere to a study protocol. Virological failure was not usually reported beyond 96 weeks, which limits the ability to extrapolate the results to the long-term success of this approach. PI monotherapy may not be as successful in patients with a history of treatment failure. The EARNEST study evaluated lopinavir monotherapy in patients who had failed first-line cART in resource-limited settings and found that 18% developed intermediate or high-level lopinavir resistance.^16^ Furthermore, intensification to cART following virological failure would only be possible if the patient could tolerate cART, which may not be the case in a real-world clinical setting. We conducted a review of patients who had received PI monotherapy at our centre in order to determine the clinical effectiveness of this strategy, the emergence of resistance and factors associated with virological failure. We show that virological failure and emergent protease mutations are more common in our cohort than in randomized controlled trials and other observational studies of European clinical settings.

## Methods

### Study design

A retrospective analysis of 10 years of prospectively collected data was conducted at a large HIV treatment centre in central London, UK. The study population was selected from HIV-1-positive patients attending the clinic between 1 January 2004 and 31 December 2013. Patients were included in the study if they were aged 16 years or over and had received at least 30 days of monotherapy with a boosted PI, and were excluded if they had been enrolled in a clinical trial of PI monotherapy. Data on demographics, antiretroviral treatment history, HIV RNA measurements and genotypic resistance testing were gathered from clinic records and the laboratory information management system.

### Laboratory methods

HIV-1 RNA quantification and genotyping were performed using validated in-house methods.^17^ Archived genotypic resistance reports were available from 2004 onwards. The Stanford HIV Drug Resistance Database^2^ was used to determine HIV-1 subtype, identify baseline and emergent protease resistance-associated mutations and evaluate the impact of emergent mutations.

### Primary and secondary endpoints

The primary endpoint was time from initiation of PI monotherapy to virological failure, which was defined as either after persistently detectable viraemia throughout the first 6 months of the PI monotherapy episode or virological rebound (two consecutive plasma samples with HIV RNA >50 copies/mL or a single plasma sample with HIV RNA >1000 copies/mL) at any point following viral suppression (at least one sample with HIV RNA <50 copies/mL). Other outcomes of interest were the times to: (i) viral re-suppression on PI monotherapy (viral suppression following virological failure); (ii) treatment discontinuation (switch to cART); and (iii) the emergence of protease resistance and loss of future PI therapeutic options (development of intermediate or high-level resistance to any PI was considered to be loss of that agent).

### Statistical analysis

Kaplan–Meier survival curves were plotted to describe the distribution of the times to virological failure and treatment discontinuation. The patients were stratified according to the presence of protease mutations at PI monotherapy initiation, baseline HIV RNA and the type of PI agent, to examine the effects of these factors. If patients had received consecutive periods of PI monotherapy with different PIs, i.e. changed PI agent but remained on PI monotherapy, this was considered as one continuous PI monotherapy episode for the main analyses. The individual periods of PI monotherapy with different agents were only considered separately when examining the effect of the type of PI on the virological outcome. The associations between factors and time to an event were tested using the log rank test, except when assessing type of PI, when Cox regression with a frailty term for patient was used to acknowledge the clustering of periods of different PI treatment for some patients. A univariate Cox regression analysis was performed to look for factors associated with virological failure. If any variables were significant then these would be included in a multivariate Cox regression analysis. Stata/IC 14 software (StataCorp LP, College Station, TX, USA) was used for all statistical analyses.

### Ethics

In the UK retrospectively obtained anonymized NHS data do not require patient consent.

## Results

### Study population

One hundred and twenty-three patients received PI monotherapy at the study centre during the 10 year observation period. Twenty-eight patients were excluded from the analysis as they had participated in a PI monotherapy trial where use of PI monotherapy was not driven by clinical circumstances. A total of 95 patients that had received PI monotherapy for clinical reasons remained for the final analysis. Their characteristics are shown in Table 1. The HIV-1 subtype was known for 74 patients, 40 (54%) of whom had subtype B infection. The other subtypes were A (six patients, 8% of known study subtypes), C (nine patients, 12%), CRF01_AE (eight patients, 11%), CRF02_AG (five patients, 7%) and 8% other subtypes (two patients harboured subtype D, and subtypes G, J and K were each found in one patient).

**Table 1.** Baseline characteristics

Male, *n* (%)	67 (71)
Age (years), median (IQR)	44 (38–48)
MSM, *n* (%)	46 (48)
Ethnicity, *n* (%)	
white	50 (53)
black	38 (40)
other/unknown	7 (7)
Nadir CD4 count (cells/mm^3^), median (IQR)	180 (70–250)
CD4 count at PI monotherapy initiation (cells/mm^3^), median (IQR)	455 (290–640)
Number of previous ART regimens prior to PI monotherapy, median (IQR)	4 (2–7)
ART experienced before PI monotherapy, *n* (%)	92 (97)
PI experienced before PI monotherapy, *n* (%)	77 (81)
Detectable plasma HIV RNA >50 copies/mL at start of episode, *n* (%)	40 (41)
Plasma HIV RNA at start of episode for the 40 episodes that started during viraemia (copies/mL), median (IQR)	6500 (510–46 000)
Duration of viral suppression prior to PI monotherapy for the 57 episodes that started during viral suppression (weeks), median (IQR)	352 (181–555)
Follow-up time (weeks), median (IQR)	143 (118–186)

Denominators of 95 patients and 97 episodes.

### PI monotherapy episodes

PIs used for monotherapy were darunavir, atazanavir and lopinavir, all boosted with ritonavir. Seventeen patients received two different PIs as monotherapy at separate times during the observation period and one patient received three different agents, giving a total of 114 individual periods of PI monotherapy. The PI agent was changed during an uninterrupted episode of monotherapy in 17 cases, and these were treated as continuous PI monotherapy episodes. The reasons for changing PI were not known; 12 changed PI while virally suppressed and 5 while viraemic. Overall, 97 continuous PI monotherapy episodes were included in the analysis (two patients had two non-consecutive PI monotherapy episodes each). The median episode duration was 126 weeks (IQR = 96–174). At the end of the study period, 77 PI monotherapy episodes were ongoing, and 57 of those had viral suppression at the last HIV RNA measurement of the study period. No patients were lost to follow-up.

### Primary endpoint

**Figure 1.**
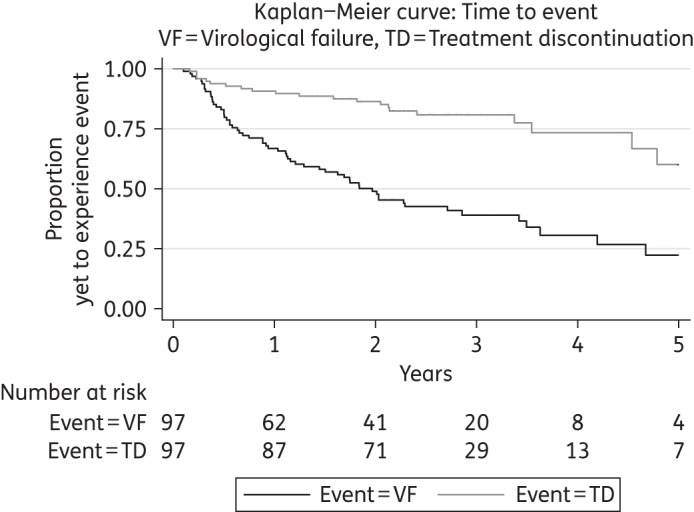
Overall treatment outcomes.

**Figure 2.**
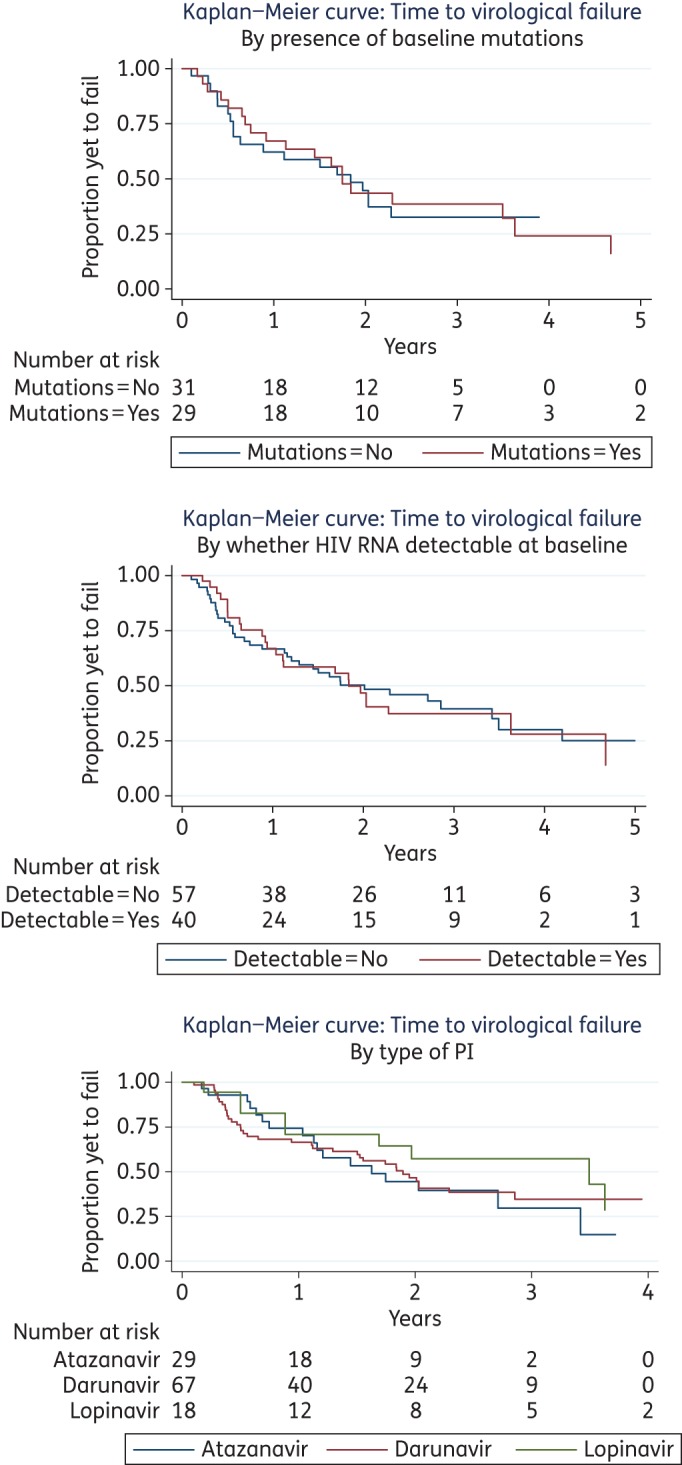
Univariate analysis of virological failure by (a) baseline protease mutations, (b) baseline plasma HIV-1 RNA viral load (detectable, >50 copies/mL; undetectable, <50 copies/mL) and (c) PI type. This figure appears in colour in the online version of *JAC* and in black and white in the print version of *JAC*.

Virological failure occurred during follow-up in 64% (62/97) of episodes. Four were due to persistent viraemia and 58 had rebounded following a period of viral suppression. The overall time-to-event analysis is shown in Figure 1. We estimate from the Kaplan–Meier curves that virological failure occurs within 2 years for around half of episodes. The presence of baseline protease mutations (*P *= 0.84), the baseline plasma HIV RNA (*P *= 0.90) and the type of PI (*P *= 0.47) did not affect the rate of virological failure (Figure 2). Univariate analyses were performed for sex, age, sexual orientation, ethnicity, nadir CD4 count, baseline CD4 count, number of previous ART regimens, ART experience, PI experience, detectable HIV RNA at PI monotherapy initiation, and duration of viral suppression prior to PI monotherapy. No variables were found to be significantly associated with virological failure and so a multivariable analysis was not performed.

### Secondary outcomes

#### Viral re-suppression

**Figure 3.**
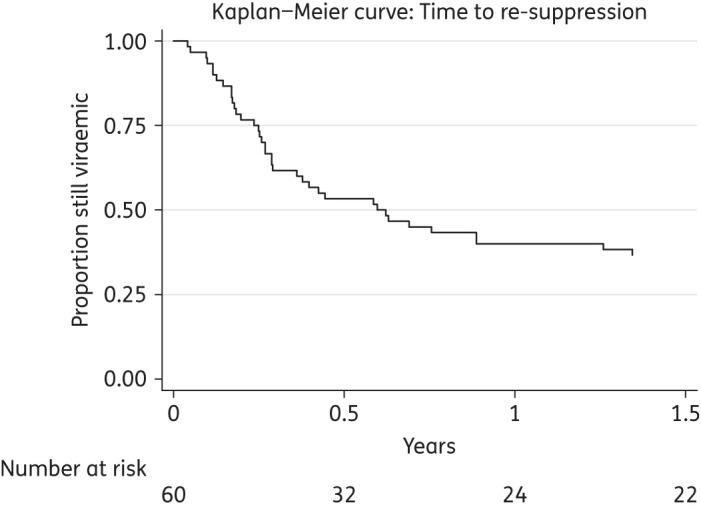
Viral re-suppression following virological failure.

Viral re-suppression on PI monotherapy, without changing therapy, occurred in 38 episodes. This represents 61% (38/62) of all virological failure episodes and 68% (38/56) of those episodes in which PI monotherapy was continued for at least 1 month following virological failure. The median time to viral re-suppression after the initial date of virological failure was 14 weeks (IQR = 9–31), as shown in Figure 3. The median level of HIV-1 RNA at virological failure was 4100 copies/mL (IQR = 760–16 500) in those patients who subsequently had viral re-suppression, compared with 3150 copies/mL (IQR = 955–23 750) in those who did not re-suppress.

#### Treatment discontinuation

Treatment was discontinued during follow-up in 21% (20/97) of PI monotherapy episodes. From Figure 1 we see that around a quarter of treatment episodes are discontinued within 4 years. In each case, the regimen was intensified to cART. There was some overlap between virological failure and treatment discontinuation, as 15 episodes during which virological failure occurred also resulted in a switch to cART. The reasons for treatment discontinuation in the remaining five episodes with sustained viral suppression were not known.

#### Genotypic protease resistance

Genotypic resistance tests performed prior to PI monotherapy were available for 60 patients, of whom 29 had pre-existing protease mutations. Five patients had major mutations at baseline and 28 had minor mutations. Thirty-six patients had tests available from both before and after starting PI monotherapy. Eight patients (8.4% of total cohort) had emergent protease mutations in repeat testing that had not been detected before PI monotherapy (Table 2). A further 14 patients had tests obtained after commencing PI monotherapy but no baseline test was available for comparison. One of these patients had protease mutations (Patient 9 in Table 2). Six patients (6.3% of total) developed intermediate or high-level resistance to at least one PI, of whom four developed intermediate or high-level resistance to the PI used for monotherapy, rendering that agent unusable for future therapy (Patients 4, 5, 8 and 9 in Table 2). Loss of future PI options resulted in a switch to cART, except for Patient 9, who had developed high-level resistance to atazanavir during atazanavir monotherapy but achieved virological suppression after switching to darunavir monotherapy. Of the five patients with pre-existing major protease mutations, two went on to develop emergent mutations (Patients 7 and 8 in Table 2). The other three patients with major baseline mutations were all PI experienced and had commenced PI monotherapy during viral suppression. One had a single major mutation (N88S) and two minor mutations (L10V and L33I) at baseline but remained virally suppressed on darunavir and then lopinavir monotherapy throughout follow-up, and the other two also had a single major mutation at baseline (M46L with minor mutations L63P, V77I/V and I93L, and L90M alone). Both experienced virological failure on atazanavir monotherapy but repeat resistance testing did not reveal any new mutations. They remained on PI monotherapy with viral suppression at the end of the study period.

**Table 2.** Patients with emergent protease mutations on PI monotherapy

No.	PI agent	HIV RNA at start (copies/mL)	Previous PI use	Protease mutations before PI monotherapy	Emergent protease mutations	Outcome of PI monotherapy	Loss of future PI options (level of resistance^a^)
1	DRV	0	none	none	minor: 10I	VF, but ongoing PI monotherapy (last RNA undetectable)	no
2	DRV	780	LPV	none	minor: 35D/G	VF, but ongoing PI monotherapy (last RNA undetectable)	no
3	DRV	350 000	ATV	none	minor: 11I/V	VF, but ongoing PI monotherapy (last RNA 100 000 copies/mL)	no
4	LPV	420	SQV	none	major: 46I, 84V minor: 10F	VF and switched to cART	ATV, FPV, IDV, NFV, SQV (high) LPV, TPV (intermediate)
5	ATV	1700	ATV	none	major: 50L, 90M minor: 71V, 73S	VF and switched to cART	ATV, NFV, SQV (high) IDV (intermediate)
6	LPV	0	SQV, NFV	minor: 10I, 58E	major: 50L	VF and switched to cART	ATV (high)
7	LPV	360	none	major: 50L, 88S minor: 33F, 71V	major: 82A/V	VF and switched to cART	IDV (intermediate)
8	DRV	0	LPV	major: 46I minor: 10F, 74A/P	major: 32I, 47V	VF and switched to cART	FPV, IDV, LPV, NFV, TPV (high) ATV, DRV (intermediate)
9	ATV then DRV	0	SQV, LPV	missing data	major: 24I, 50L minor: 71T	VF on ATV, switch to DRV ongoing PI monotherapy (last RNA undetectable)	ATV (high)

VF, virological failure; DRV, darunavir; LPV, lopinavir; ATV, atazanavir; SQV, saquinavir; NFV, nelfinavir; FPV, fosamprenavir; IDV, indinavir; TPV, timprenavir.

^a^Stanford HIV Drug Resistance Database interpretation.

## Discussion

Virological failure was common in this real-world setting and occurred in two-thirds of PI monotherapy episodes. Our patients had less favourable resistance profiles and higher HIV RNA levels at baseline than most PI monotherapy trial participants. They also had a higher incidence of emergent protease mutations and greater loss of future PI options. Despite this, over half the cohort ended the observation period continuing on PI monotherapy with viral suppression.

This is the first study, to our knowledge, to investigate the real-life use of PI monotherapy in a clinical setting where the use of PI-containing cART as first line is uncommon. There have been other observational studies in Spain and France, where PI-containing cART is more frequently used as first-line treatment, and our cohort otherwise had comparable baseline characteristics to those studies, with a preponderance of male patients, white ethnicity, MSM mode of transmission and subtype B infection, all typical of a European setting.^18–22^ Another similarity was that over three-quarters of patients were PI experienced prior to receiving monotherapy. Our cohort had a median baseline CD4 count of 455 cells/mm,^3^ lower than the averages reported in the other clinical studies, which were between 541 and 608.^18,19,21–23^ The median nadir CD4 count of 180 was within the range found in the clinical studies (medians were between 155 and 238).^18–21,23^ We did not find an independent association between CD4 count and failure, as did four of the observational studies.^18,20,21,23^ One study found an association between a nadir CD4 of <200 cells/mm^3^ and virological failure.^19^ Two of the trials, PROTEA^14^ and MODAt,^15^ found a low CD4 count was related to outcome, while MONET^7^ did not find an independent association.

There were some clear differences between our findings and the other analyses of PI monotherapy in clinical practice. In the other studies, plasma HIV RNA had to be fully suppressed for at least 6 months before PI monotherapy was commenced, whereas ∼40% of our patients were viraemic at PI monotherapy initiation.^18–23^ We only investigated patients prescribed PI monotherapy for clinical reasons but one large multicentre study of clinical practice did not exclude PI monotherapy trial participants.^19^ When the indications for PI monotherapy were cited they were commonly treatment simplification or patient preference.^18,21–23^ This is in line with European and Spanish guidelines, which since 2009 cite PI monotherapy as an alternative strategy in the management of selected patients.^24,25^ French national guidelines have been more conservative, in 2010 deeming PI monotherapy to be inadequate, and in the 2013 iteration indicating that this strategy can be considered on a case-by-case basis.^26,27^ However, UK guidelines either did not mention PI monotherapy or expressly recommended against its use throughout the 10 year period applicable to the present study.^28^ PI monotherapy is not generally considered an acceptable treatment option in the UK unless there are extenuating circumstances that preclude the use of cART.^29^ It is our experience that such scenarios where PI monotherapy is employed include severely ill patients receiving intensive care who require a regimen with minimal systemic toxicity to control viral replication,^30^ or a patient struggling to comply with a more complex regimen where PI monotherapy is used as a holding strategy while further support and adherence interventions are attempted. A limitation of this study is the lack of complete data on the reasons for PI monotherapy and subsequent management decisions. However, we believe these reflect practical choices related to adherence and toxicity issues.

Previous clinical practice studies reported 48 week virological failure rates of 4%,^22^ 10%,^21^ 14%^18^ and 31%.^20^ The cumulative proportion of episodes with virological failure in this study was around 30% by 48 weeks and around 50% by 2 years. A reason for the higher risk of virological failure than in most other studies, particularly in comparison with trial participants, may be that our cohort was less likely to adhere to therapy. Virological re-suppression was common in our study when patients remained on PI monotherapy following failure, suggesting that adherence was the main determinant of loss of viral control, and not lack of drug potency. The HIV-1 RNA level at virological failure did not differ significantly between those who did and did not go on to experience viral re-suppression. The phenomenon of re-suppression has also been observed amongst first-line cART-treated patients in resource-limited settings.^31^ Continuation of monotherapy despite virological failure at our centre probably reflects a pragmatic clinical decision based on concerns about adherence and dosing/toxicity issues associated with alternative regimens.

In the three clinical studies reporting baseline genotypes, the prevalence of major protease mutations was between 3% and 7% prior to PI monotherapy, similar to the 5% baseline prevalence in this study.^19,21,22^ There was considerable variation in the frequency of genotypic resistance testing following virological failure. Between 20% and 66% of virological failure patients in the clinical studies had subsequent resistance testing, compared with 80% in our study. There were also differences in the reporting of mutations across studies. These two factors complicate comparisons of prior estimates of emergent mutations in clinical settings, which ranged from 0.4% to 2.2%, with our data (8%).^18–23^ There was no significant difference in study endpoints when the cohort was stratified according to baseline protease mutations, HIV RNA at initiation or PI agent. Two clinical practice studies found that a shorter period of virological suppression prior to PI monotherapy was associated with increased risk of virological failure.^19,20^ One of these also found that virological failure and treatment failure were more likely to occur in patients receiving atazanavir monotherapy, compared with those on lopinavir or darunavir.^20^ Another found lopinavir was associated with a greater risk of treatment discontinuation than darunavir; however, the follow-up period was much longer in this group.^18^ A further study of similar size to ours did not find any significant predictors of virological failure.^21^ One limitation of the present study is the relatively small sample size that could have resulted in insufficient statistical power to detect small differences among subgroups. However, our findings are in line with a large Spanish study, which did not find any significant effect of minor pre-existing mutations, PI agent or duration of previous viral suppression on the rates of virological failure in patients without major baseline protease mutations commencing either darunavir or lopinavir monotherapy.^23^

An advantage of the present study is that it investigates a real-world clinical setting with a pragmatic approach to the use of PI monotherapy in patients with less favourable baseline characteristics. However, these same attributes could limit the external validity for other settings in which PI monotherapy is to be used in stable patients with virological suppression and a good history of treatment engagement. We found high rates of virological failure but that if PI monotherapy was continued then virological re-suppression was often achieved. Emergent resistance was also more common than previously reported, as was the loss of future PI options. Despite this, the majority of PI monotherapy episodes were ongoing at the end of this study and over half were ongoing with viral suppression. Some of the discrepancies in outcomes compared with other studies may be a result of a longer observation period, thus capturing more events. It may be that prolonged follow-up of several years is required to understand the true outcomes of PI monotherapy in a clinical setting.

## Funding

This work was funded by the Wellcome Trust, fellowships to R. K. G. (WT093722MA) and K. E. B. (170461), and the British Infection Society, fellowship to D. C. (E19115).

We also received support from the National Institutes for Health Research University College London Hospitals Biomedical Research Centre.

## Transparency declarations

K. E. B., D. C., E. N. and A. J. C. have no conflicts of interest. R. F. M. has received honoraria from Gilead, Janssen, Merck and ViiV for giving non-promotional lectures on clinical aspects of HIV, is a member of the British HIV Association TB/HIV Guidelines Committee and is a panel member for Guidelines for the Prevention and Treatment of Opportunistic Infections in HIV-Infected Adults and Adolescents National Institutes of Health, Centers for Disease Control and Prevention, HIV Medicine Association of the Infectious Diseases Society of America. R. K. G. has received honoraria from Janssen for giving non-promotional lectures on HIV drug resistance.
